# Tamoxifen induces fatty liver disease in breast cancer through the MAPK8/FoxO pathway

**DOI:** 10.1002/ctm2.5

**Published:** 2020-04-23

**Authors:** Liuyun Gong, Hanmin Tang, Zhenzhen Luo, Xiao Sun, Xinyue Tan, Lina Xie, Yutiantian Lei, Mengjiao Cai, Chenchen He, Jinlu Ma, Suxia Han

**Affiliations:** ^1^ Department of Oncology The First Affiliated Hospital Xi'an Jiaotong University Xi'an PR China

**Keywords:** bioinformatics analysis, breast cancer, fatty liver, FoxO signaling pathway, MAPK8, TAM

## Abstract

**Background:**

Prevention of metabolic complications of long‐term adjuvant endocrine therapy in breast cancers remained a challenge. We aimed to investigate the molecular mechanism in the development of tamoxifen (TAM)‐induced fatty liver in both estrogen receptor (ER)‐positive and ER‐negative breast cancer.

**Methods and results:**

First, the direct protein targets (DPTs) of TAM were identified using DrugBank5.1.7. We found that mitogen‐activated protein kinase 8 (MAPK8) was one DPT of TAM. We identified significant genes in breast cancer and fatty liver disease (FLD) using the MalaCards human disease database. Next, we analyzed the Kyoto Encyclopedia of Genes and Genomes (KEGG) pathways of those significant genes in breast cancer and FLD using the Search Tool for the Retrieval of Interacting Genes/Proteins (STRING). We found that overlapping KEGG pathways in these two diseases were MAPK signaling pathway, Forkhead box O (FoxO) signaling pathway, HIF‐1 signaling pathway, AGE‐RAGE signaling pathway in diabetic complications, and PI3K‐Akt signaling pathway. Furthermore, the KEGG Mapper showed that the MAPK signaling pathway was related to the FoxO signaling pathway. Finally, the functional relevance of breast cancer and TAM‐induced FLD was validated by Western blot analysis. We verified that TAM may induce fatty liver in breast cancer through the MAPK8/FoxO signaling pathway.

**Conclusion:**

Bioinformatics analysis combined with conventional experiments may improve our understanding of the molecular mechanisms underlying side effects of cancer drugs, thereby making this method a new paradigm for guiding future studies on this issue.

## 1 BACKGROUND

Breast cancer is the most common cancer in women and the main cause of cancer‐related death in women worldwide.^1^ Recently, obesity has been regarded as a risk factor for this disease, and fatty liver disease (FLD) and breast cancer have been found to share similar risk factors, including obesity and metabolic abnormalities. Hyperinsulinemia is also associated with both FLD and breast cancer, suggesting there is a mechanistic link between the two diseases.^2^

Tamoxifen (TAM) is used for the treatment of breast cancer widely.^3^ It is noticeable, however, that hepatocyte steatosis has been described in studies of patients with breast cancer because of TAM,^4^, ^5^ and TAM is known to induce this condition in half of the patients within the first 2 years of TAM treatment.^6^, ^7^, ^8^ Therapeutic intervention to prevent TAM‐induced hepatocyte steatosis may improve the safety of TAM usage.^9^ Thus, there is an urgent need to find effective paradigms to clarify the functional mechanisms underlying breast cancer and TAM‐induced FLD.

In recent years, tumor databases and drug databases have developed and are continuously improving, especially drug databases, which combine drug action information and drug target genes are rapidly developing.^10^ Integrative analysis of tumor databases and drug databases derives a good technique to discover the mechanism underlying drug‐induced diseases.^11^, ^12^, ^13^

In this study, we identified direct protein targets (DPTs) of TAM using DrugBank5.1.7. We found that mitogen‐activated protein kinase 8 (MAPK8) was one DPT of TAM. Meanwhile, we identified significant genes in breast cancer and FLD using the MalaCards human disease database, and the results of Kyoto Encyclopedia of Genes and Genomes (KEGG) analysis showed that the MAPK and Forkhead box O (FoxO) signaling pathways were related to both breast cancer and FLD. Further, the KEGG Mapper showed that the MAPK signaling pathway was upstream of FoxO signaling pathway. Finally, we explored the functional relevance of TAM‐induced fatty liver in breast cancer with the MTT assay, colony formation assay, flow cytometry, and Western blotting. The result showed that TAM may induce fatty liver in patients with breast cancer by interfering with the MAPK8/FoxO signaling pathway.

## 2 MATERIALS AND METHODS

### 2.1 Recognition of DPTs of TAM

The DrugBank (https://www.drugbank.ca) is a rich database that combines drug interaction information and drug target genes. It has been widely used for drug research since 2006.^10^ Manual literature searches for data are guided by PolySearch2, a text‐mining tool developed for DrugBank annotation projects.^14^ The DPTs of TAM were driven from DrugBank by inputting TAM in the search box and clicking Targets.

### 2.2 Identification of differentially expressed DPTs of TAM

The Gene Expression Profiling Interactive Analysis (GEPIA) (http://gepia.cancer-pku.cn/) is a tool. It is based on The Cancer Genome Atlas and GTEx data and delivers fast and customizable functionalities. There are rich functions including differential expression analysis, similar gene detection, correlation analysis, and patient survival analysis in GEPIA.^15^ First, a DPT in the search box was inputted and GoPIA! was clicked in GEPIA, then the cancer type with breast cancer (BRCA) was chosen, and finally, the differential expression of DPT of TAM was identified.

### 2.3 Analysis of significant genes in breast cancer and FLD

MalaCards (http://www.malacards.org/) is a database of human diseases and their annotations, whose architecture and strategy is based on the GeneCards database. MalaCards generates a web card for more than 20 000 human diseases in six global categories.^16^ When searched for breast cancer and fatty liver in the MalaCards, a table containing significant genes of breast cancer and fatty liver can be downloaded directly. Cytoscape is one of the most successful network biology analysis and visualization tools.^17^ The significant genes of breast cancer and fatty liver were visualized using Cytoscape 3.7.1.

### 2.4 Analysis of KEGG pathways in breast cancer and fatty liver

Search tool for the Retrieval of Interacting Genes (STRING) (https://string-db.org/cgi/input.pl) is a public web‐based tool that can evaluate the protein‐protein interaction network, KEGG pathways, and gene ontology terms.^18^, ^19^ We analyzed KEGG pathways in the significant genes of breast cancer and FLD using STRING. When those significant genes were searched (with organism being *Homo sapiens*), the analysis result showed the KEGG pathways in breast cancer and FLD. And the result was visualized using OriginPro 2015. KEGG Mapper is a suite of KEGG mapping tools available at the KEGG website (https://www.kegg.jp/ or https://www.genome.jp/kegg/); we mapped MAPK signaling pathway and FoxO signaling pathway using this tool.

### 2.5 Cell culture and reagents

Both the human breast cancer cell lines and the human liver cell lines were obtained from The American Type Culture Collection (Manassas, VA). MCF‐7, MDA‐MB‐231, and LO2 cells were cultured under standard cell culture conditions in Dulbecco's Modified Eagle's Medium containing 10% serum at 37°C in a humidified atmosphere with 5% CO_2_. T47D and ZR‐75 cells were cultured under standard cell culture conditions in RPMI‐1640 medium containing 10% serum at 37°C in a humidified atmosphere with 5% CO_2_. TAM (C_26_H_29_NO; molecular weight: 371.51) purchased from MedChemExpress (MCE) was dissolved in dimethyl sulfoxide (DMSO) at the stock concentration of 27 mmol/L initially. MTT (3‐(4,5‐dimethyl‐2‐thiazolyl)‐2,5‐diphenyl‐2H‐tetrazolium bromide) and Oil Red O were purchased from Sigma‐Aldrich (St. Louis, MO). Triglyceride Assay Kit was purchased from Jiancheng (Nanjing, China). Antibodies were purchased from Cell Signaling Technology (Danvers, MA) and Proteintech Group Inc. (Rosemont, IL).

### 2.6 Cell viability assay

Cancer cell lines (MCF‐7, T47D, ZR‐75, and MDA‐MB‐231) were plated in 96‐well plates at a density of 1 × 10^3^ cells per well and allowed to adhere overnight, and then treated at various concentrations (0, 5, 10, 20, 30, and 40 µmol/L) of TAM. At the indicated time points (0, 12, 24, and 36 hours), cell viability was assessed by the MTT assay and was measured using a multiwell microplate reader (BIO‐TEC Inc., Richmond, VA) at an absorbance of 490 nm.

### 2.7 Colony formation assay

A total of 1000 cells in the control group and 20 000 cells in the drug group were seeded into six‐well cell culture clusters and allowed to adhere overnight. Then TAM was added to the cells for 24 hours, after which media was replaced with drug‐free media. Cells were cultured for an additional 10 days to allow the colonies to form. At the related time points, colonies were fixed in 4% paraformaldehyde and then stained with 0.1% crystal violet solution, rinsed, and imaged. The number of colonies >0.5 mm in diameter was counted using a microscope (Nikon Eclipse Ti‐S, Tokyo, Japan) at a magnification of 20× and 40×.

### 2.8 Apoptosis assay

Cell apoptosis was assessed by flow cytometry with PE Annexin V Apoptosis Detection Kit I (Becton Dickinson Biosciences, Franklin Lakes, NJ) according to the manufacturer's instructions. Briefly, cancer cells were seeded in 6‐well plates at a density of 1 × 10^5^ cells per well. After being starved overnight, cells were treated with fresh medium containing various concentrations of TAM for 24 hours. Then cells were trypsinized, washed with phosphate‐buffered saline (PBS), and stained with PE Annexin V. The percentage of apoptotic cells was quantified by flow cytometry using a FACSCalibur instrument (BD Biosciences). The total apoptosis rate was calculated by summing the rate of early apoptotic cells (7‐AAD−/PE Annexin V+) and late apoptotic cells (7‐AAD+/PE Annexin V+).

### 2.9 Oil Red O Staining

LO2 cells were grown in 6‐well cell culture clusters and treated at various concentrations (0, 5, 10, 20, 30, and 40 µmol/L) of TAM after 24 hours. Then they were washed with PBS and fixed in paraformaldehyde solution for 10 minutes at room temperature. After fixation, cells were gently washed with ddH2O and stained with a working solution of 0.5 g Oil Red O for 30 minutes. The stained hepatocytes were washed three times with PBS to remove the unincorporated dye, and then examined by laser scanning confocal microscopy.

### 2.10 Triglyceride measurement

LO2 cells were preincubated in a 6 cm cell culture dish for 24 hours and then cultured in DMEM with TAM (0, 10, 15, 20, 30, and 40 µmol/L). After 24 hours of incubation, cells were transferred into an Eppendorf tube (1.5 mL) and centrifuged at 800 rpm for 5 minutes. Cell pellets were washed with PBS and centrifuged again at 800 rpm for 5 minutes. Total triglyceride (TG) was extracted by RIPA Lysis Buffer (Fisher, Pittsburgh, PA). The concentration of TG was determined using the TG Assay Kit (Jiancheng, Nanjing, China) and normalized by protein concentration according to the manufacturer's instructions.

### 2.11 Western blot analysis

Total proteins were extracted by RIPA Lysis Buffer and their concentration was determined using the BCA Protein Assay Kit (Pierce, Rockford, IL) according to the manufacturer's instructions. Then Western blotting was performed. The 4‐12% Bis‐Tris precast gels (Bio‐Rad, Hercules, CA) were used for electrophoresis. Equal volumes of cell total protein were loaded and subsequently electrotransferred to a nitrocellulose membrane. The membrane was blocked in 5% non‐fat milk (Lab Scientific, Livingston, NJ), followed by incubation with primary and horseradish peroxidase–conjugated secondary antibodies overnight and 2 hours, respectively.^20^, ^21^, ^22^, ^23^, ^24^, ^25^, ^26^ Protein expression was visualized by enhanced chemiluminescence (GE, Buckinghamshire, UK). Images were captured using the ChemiDoc XRS imaging system (Bio‐Rad), and Quantity One image software was used for densitometry analysis of each band. GAPDH was used as the internal loading control.

### 2.12 Statistics

The results are expressed as the mean ± SD. The lipid accumulation in LO2 cells with different TAM concentrations was analyzed by analysis of variance using GraphPad Prism 6.0. Other data were analyzed by the Student's *t*‐test using GraphPad Prism 6.0. *P* values <0.05 were considered to be statistically significant. Each experiment was performed at least three times.

## 3 RESULTS

### 3.1 Bioinformatics analysis of TAM, breast cancer, and FLD

**FIGURE 1:**
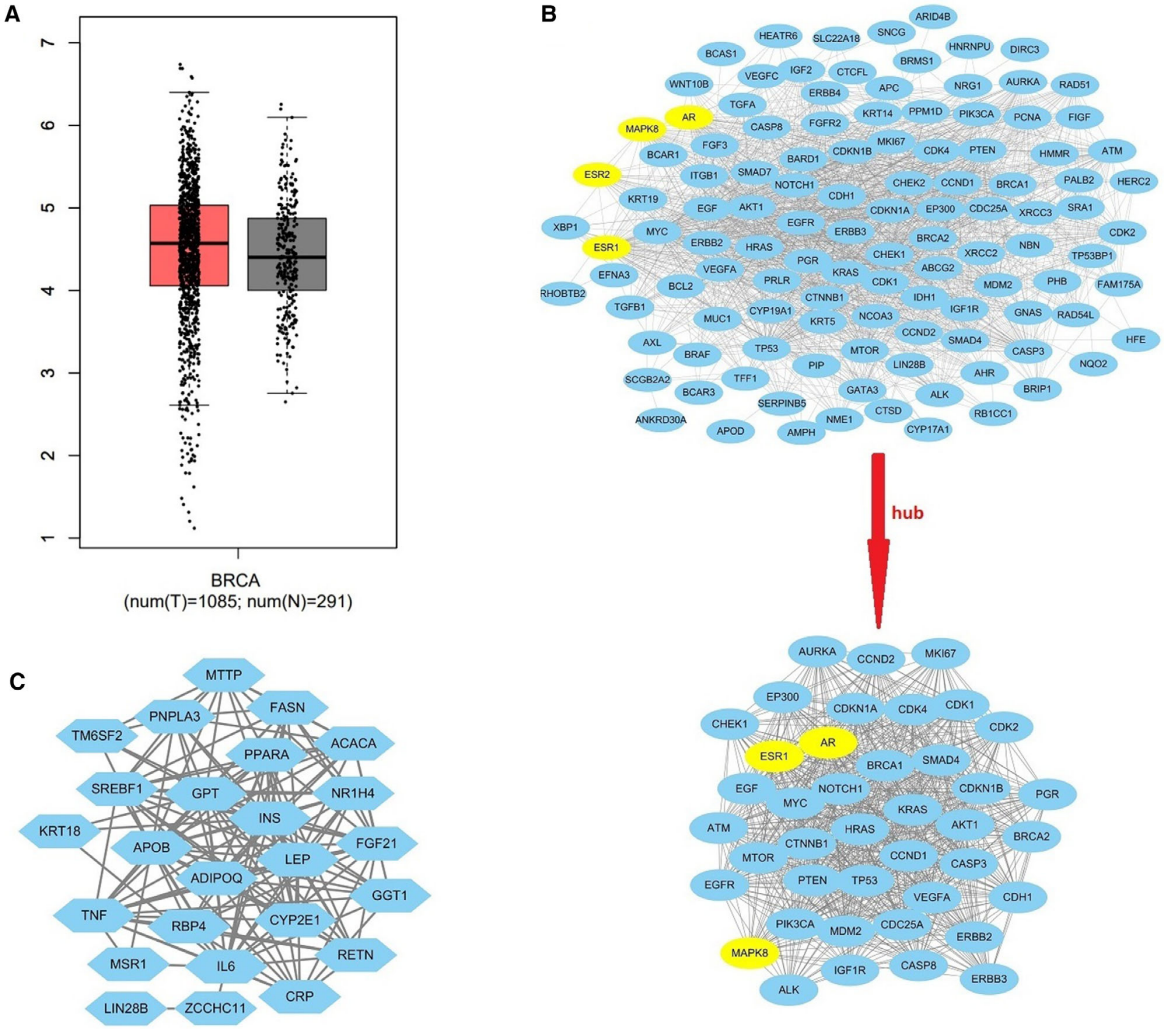
The different expression of MAPK8 in breast cancer samples to normal samples. A, The red box shows breast cancer samples and the black box shows normal samples. B, Significant genes and hub genes of breast cancer, and the yellow nodes were DPT of TAM. C, Significant genes of fatty liver

TAM was output as DB00675 (APRD00123) from DrugBank 5.1.4 with 17 primary DPTs (Table 1). It is noteworthy that MAPK8 was overexpressed in breast cancer samples compared to normal samples (Figure 1A). Significant genes and 41 hub genes in breast cancer were identified (Figure 1B). Significant genes in FLD are shown in Figure 1C.

**TABLE 1:** Identification of direct targets of tamoxifen using DrugBank

Searched drug (1/1)		Target (17)	
Name	Target symbol	Uniprot ID	Uniprot name
Tamoxifen	ESR2	Q92731	Estrogen receptor beta
	ESR1	P03372	Estrogen receptor alpha
	MAPK8	P45983	Mitogen‐activated protein kinase 8
	SHBG	P04278	Sex hormone‐binding globulin
	ESRRG	P62508	Estrogen‐related receptor gamma
	NR1I2	O75469	Nuclear receptor subfamily 1 group I member 2
	KCNH2	Q12809	Potassium voltage‐gated channel subfamily H member 2
	AR	P10275	Androgen receptor
	EBP	Q15125	3‐beta‐Hydroxysteroid‐Delta(8),Delta(7)‐isomerase
	Protein group	Q05513	Protein kinase C zeta type
		Q04759	Protein kinase C theta type
		P41743	Protein kinase C iota type
		P05129	Protein kinase C gamma type
		Q02156	Protein kinase C epsilon type
		Q05655	Protein kinase C delta type
		P05771	Protein kinase C beta type
		P17252	Protein kinase C alpha type

**FIGURE 2:**
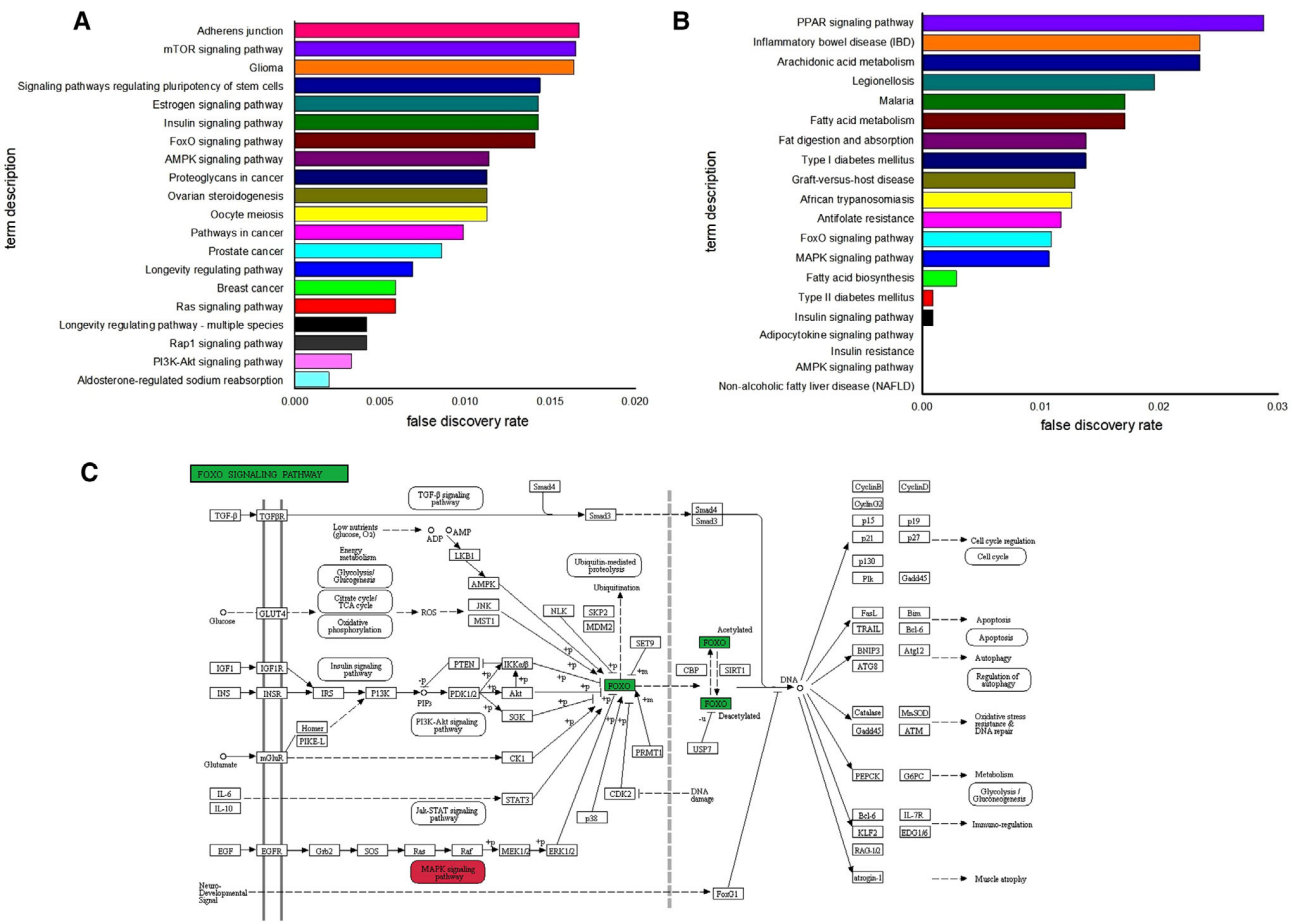
A, Top 20 KEGG pathways of breast cancer. B, Top 20 KEGG pathways of fatty liver. C, KEGG map of FoxO signaling pathway

The results of KEGG analysis of breast cancer are shown in Table 2, and the top 20 KEGG pathways in breast cancer are shown in Figure 2A. The results of KEGG analysis of FLD are shown in Table 3, and the top 20 KEGG pathways are shown in Figure 2B. The five overlapping KEGG pathways in both breast cancer and FLD were the phosphoinositide 3‐kinase‐Akt, FoxO, MAPK, hypoxia inducible factor‐1, and advanced glycation end product receptor for advanced glycation end product (in diabetic complications) signaling pathways. Meanwhile, KEGG mapper (Figure 2C) showed that the MAPK signaling pathway was upstream of the FoxO signaling pathway.

**TABLE 2:** Top 50 KEGG pathway in breast cancer

Term ID	Term description	False discovery rate	Matching proteins in your network (labels)
hsa05200	Pathways in cancer	1.04E‐38	AKT1,ALK,APC,AR,BCL2,BRAF,BRCA2,CASP3, CASP8,CCND1,CCND2,CDH1,CDK2,CDK4,CDKN1A, CDKN1B,CTNNB1,EGF,EGFR,EP300,ERBB2,ESR1, ESR2,FGF3,FGFR2,FIGF,GNAS,HRAS,IGF1R,IGF2, ITGB1,KRAS,MAPK8,MDM2,MTOR,MYC,NCOA3, NOTCH1,PIK3CA,PTEN,SMAD4,TGFA,TGFB1,TP53, VEGFA,VEGFC,WNT10B
hsa05224	Breast cancer	1.50E‐28	AKT1,APC,BRAF,BRCA1,BRCA2,CCND1,CDK4,CDKN1A, CTNNB1,EGF,EGFR,ERBB2,ESR1,ESR2,FGF3,HRAS,IGF1R, KRAS,MTOR,MYC,NCOA3,NOTCH1,PGR,PIK3CA,PTEN, TP53,WNT10B
hsa04151	PI3K‐Akt signaling pathway	4.37E‐27	AKT1,BCL2,BRCA1,CCND1,CCND2,CDK2,CDK4,CDKN1A, CDKN1B,EFNA3,EGF,EGFR,ERBB2,ERBB3,ERBB4,FGF3, FGFR2,FIGF,HRAS,IGF1R,IGF2,ITGB1,KRAS,MDM2,MTOR, MYC,PIK3CA,PRLR,PTEN,TGFA,TP53,VEGFA,VEGFC
hsa05206	MicroRNAs in cancer	4.37E‐27	APC,ATM,BCL2,BRCA1,CASP3,CCND1,CCND2,CDC25A, CDKN1A,CDKN1B,EFNA3,EGFR,EP300,ERBB2,ERBB3, HRAS,KRAS,MDM2,MTOR,MYC,NOTCH1,PIK3CA,PTEN,S ERPINB5,TP53,VEGFA
hsa05215	Prostate cancer	1.01E‐26	AKT1,AR,BCL2,BRAF,CCND1,CDK2,CDKN1A,CDKN1B, CTNNB1,EGF,EGFR,EP300,ERBB2,FGFR2,HRAS,IGF1R, KRAS,MDM2,MTOR,PIK3CA,PTEN,TGFA,TP53
hsa01522	Endocrine resistance	2.14E‐25	AKT1,BCL2,BRAF,CCND1,CDK4,CDKN1A,CDKN1B, EGFR,ERBB2,ESR1,ESR2,GNAS,HRAS,IGF1R,KRAS, MAPK8,MDM2,MTOR,NCOA3,NOTCH1,PIK3CA,TP53
hsa05226	Gastric cancer	1.23E‐24	AKT1,APC,BCL2,BRAF,CCND1,CDH1,CDK2,CDKN1A, CDKN1B,CTNNB1,EGF,EGFR,ERBB2,FGF3,FGFR2, HRAS,KRAS,MTOR,MYC,PIK3CA,SMAD4,TGFB1, TP53,WNT10B
hsa05210	Colorectal cancer	2.96E‐23	AKT1,APC,BCL2,BRAF,CASP3,CCND1,CDKN1A, CTNNB1,EGF,EGFR,HRAS,KRAS,MAPK8,MTOR,MYC, PIK3CA,SMAD4,TGFA,TGFB1,TP53
hsa05205	Proteoglycans in cancer	4.23E‐22	AKT1,BRAF,CASP3,CCND1,CDKN1A,CTNNB1,EGFR, ERBB2,ERBB3,ERBB4,ESR1,HRAS,IGF1R,IGF2,ITGB1, KRAS,MDM2,MTOR,MYC,PIK3CA,TGFB1,TP53,VEGFA, WNT10B
hsa05212	Pancreatic cancer	3.41E‐21	AKT1,BRAF,BRCA2,CCND1,CDK4,CDKN1A,EGF,EGFR, ERBB2,KRAS,MAPK8,MTOR,PIK3CA,SMAD4,TGFA, TGFB1,TP53,VEGFA
hsa05165	Human papillomavirus infection	3.57E‐21	AKT1,APC,ATM,CASP3,CASP8,CCND1,CCND2,CDK2, CDK4,CDKN1A,CDKN1B,CTNNB1,EGF,EGFR,EP300, GNAS,HRAS,ITGB1,KRAS,MDM2,MTOR,NOTCH1, PIK3CA,PTEN,TP53,VEGFA,WNT10B
hsa01521	EGFR tyrosine kinase inhibitor resistance	6.48E‐21	AKT1,AXL,BCL2,BRAF,EGF,EGFR,ERBB2,ERBB3, FGFR2,HRAS,IGF1R,KRAS,MTOR,NRG1,PIK3CA, PTEN,TGFA,VEGFA
hsa04068	FoxO signaling pathway	3.36E‐20	AKT1,ATM,BRAF,CCND1,CCND2,CDK2,CDKN1A, CDKN1B,EGF,EGFR,EP300,HRAS,IGF1R,KRAS, MAPK8,MDM2,PIK3CA,PTEN,SMAD4,TGFB1
hsa04218	Cellular senescence	3.58E‐20	AKT1,ATM,CCND1,CCND2,CDC25A,CDK1,CDK2, CDK4,CDKN1A,CHEK1,CHEK2,HRAS,KRAS,MDM2, MTOR,MYC,NBN,PIK3CA,PTEN,TGFB1,TP53
hsa05225	Hepatocellular carcinoma	7.66E‐20	AKT1,APC,BRAF,CCND1,CDK4,CDKN1A,CTNNB1, EGFR,HRAS,IGF1R,IGF2,KRAS,MTOR,MYC,PIK3CA, PTEN,SMAD4,TGFA,TGFB1,TP53,WNT10B
hsa05213	Endometrial cancer	9.33E‐20	AKT1,APC,BRAF,CCND1,CDH1,CDKN1A,CTNNB1, EGF,EGFR,ERBB2,HRAS,KRAS,MYC,PIK3CA,PTEN,TP53
hsa05161	Hepatitis B	1.25E‐19	AKT1,BCL2,CASP3,CASP8,CCND1,CDK2,CDK4,CDKN1A, CDKN1B,EP300,HRAS,KRAS,MAPK8,MYC,PCNA,PIK3CA, PTEN,SMAD4,TGFB1,TP53
hsa04012	ErbB signaling pathway	3.87E‐19	AKT1,BRAF,CDKN1A,CDKN1B,EGF,EGFR,ERBB2, ERBB3,ERBB4,HRAS,KRAS,MAPK8,MTOR,MYC,NRG1, PIK3CA,TGFA
hsa04115	p53 Signaling pathway	7.09E‐19	ATM,CASP3,CASP8,CCND1,CCND2,CDK1,CDK2,CDK4, CDKN1A,CHEK1,CHEK2,MDM2,PPM1D,PTEN,SERPINB5, TP53
hsa05214	Glioma	7.09E‐19	AKT1,BRAF,CCND1,CDK4,CDKN1A,EGF,EGFR,HRAS, IGF1R,KRAS,MDM2,MTOR,PIK3CA,PTEN,TGFA,TP53
hsa05218	Melanoma	1.43E‐18	AKT1,BRAF,CCND1,CDH1,CDK4,CDKN1A,EGF,EGFR, FGF3,HRAS,IGF1R,KRAS,MDM2,PIK3CA,PTEN,TP53
hsa05219	Bladder cancer	1.63E‐18	BRAF,CCND1,CDH1,CDK4,CDKN1A,EGF,EGFR,ERBB2, HRAS,KRAS,MDM2,MYC,TP53,VEGFA
hsa04110	Cell cycle	4.82E‐18	ATM,CCND1,CCND2,CDC25A,CDK1,CDK2,CDK4, CDKN1A,CDKN1B,CHEK1,CHEK2,EP300,MDM2, MYC,PCNA,SMAD4,TGFB1,TP53
hsa05166	HTLV‐I infection	9.96E‐18	AKT1,APC,ATM,CCND1,CCND2,CDK4,CDKN1A, CHEK1,CHEK2,CTNNB1,EP300,HRAS,KRAS,MAPK8, MYC,PCNA,PIK3CA,SMAD4,TGFB1,TP53,WNT10B,XBP1
hsa04010	MAPK signaling pathway	1.55E‐17	AKT1,BRAF,CASP3,EFNA3,EGF,EGFR,ERBB2,ERBB3, ERBB4,FGF3,FGFR2,FIGF,HRAS,IGF1R,IGF2,KRAS, MAPK8,MYC,TGFA,TGFB1,TP53,VEGFA,VEGFC
hsa05223	Nonsmall cell lung cancer	4.20E‐16	AKT1,ALK,BRAF,CCND1,CDK4,CDKN1A,EGF, EGFR,ERBB2,HRAS,KRAS,PIK3CA,TGFA,TP53
hsa04510	Focal adhesion	5.04E‐16	AKT1,BCAR1,BCL2,BRAF,CCND1,CCND2,CTNNB1, EGF,EGFR,ERBB2,FIGF,HRAS,IGF1R,ITGB1,MAPK8, PIK3CA,PTEN,VEGFA,VEGFC
hsa04015	Rap1 signaling pathway	8.13E‐16	AKT1,BCAR1,BRAF,CDH1,CTNNB1,EFNA3,EGF, EGFR,FGF3,FGFR2,FIGF,GNAS,HRAS,IGF1R,ITGB1, KRAS,PIK3CA,VEGFA,VEGFC
hsa04933	AGE‐RAGE signaling pathway in diabetic complications	2.09E‐15	AKT1,BCL2,CASP3,CCND1,CDK4,CDKN1B,FIGF, HRAS,KRAS,MAPK8,PIK3CA,SMAD4,TGFB1, VEGFA,VEGFC
hsa05220	Chronic myeloid leukemia	2.09E‐15	AKT1,BRAF,CCND1,CDK4,CDKN1A,CDKN1B, HRAS,KRAS,MDM2,MYC,PIK3CA,SMAD4,TGFB1,TP53
hsa04915	Estrogen signaling pathway	6.00E‐15	AKT1,BCL2,CTSD,EGFR,ESR1,ESR2,GNAS,HRAS, KRAS,KRT14,KRT19,NCOA3,PGR,PIK3CA,TFF1,TGFA
hsa03440	Homologous recombination	7.23E‐14	ATM,BARD1,BRCA1,BRCA2,BRIP1,FAM175A,NBN, PALB2,RAD54L,XRCC2,XRCC3
hsa05230	Central carbon metabolism in cancer	2.66E‐13	AKT1,EGFR,ERBB2,FGFR2,HRAS,IDH1,KRAS, MTOR,MYC,PIK3CA,PTEN,TP53
hsa04919	Thyroid hormone signaling pathway	3.24E‐13	AKT1,CCND1,CTNNB1,EP300,ESR1,HRAS,KRAS, MDM2,MTOR,MYC,NCOA3,NOTCH1,PIK3CA,TP53
hsa05222	Small cell lung cancer	4.60E‐13	AKT1,BCL2,CASP3,CCND1,CDK2,CDK4,CDKN1A, CDKN1B,ITGB1,MYC,PIK3CA,PTEN,TP53
hsa05167	Kaposi's sarcoma‐associated herpesvirus infection	5.10E‐13	AKT1,CASP3,CASP8,CCND1,CDK4,CDKN1A, CTNNB1,EP300,HRAS,KRAS,MAPK8,MTOR, MYC,PIK3CA,TP53,VEGFA
hsa05203	Viral carcinogenesis	5.10E‐13	CASP3,CASP8,CCND1,CCND2,CDK1,CDK2, CDK4,CDKN1A,CDKN1B,CHEK1,EP300,HRAS, KRAS,MDM2,PIK3CA,TP53
hsa04014	Ras signaling pathway	1.13E‐11	AKT1,EFNA3,EGF,EGFR,FGF3,FGFR2,FIGF, HRAS,IGF1R,IGF2,KRAS,MAPK8,PIK3CA,TGFA, VEGFA,VEGFC
hsa04917	Prolactin signaling pathway	1.13E‐11	AKT1,CCND1,CCND2,CYP17A1,ESR1,ESR2, HRAS,KRAS,MAPK8,PIK3CA,PRLR
hsa01524	Platinum drug resistance	1.26E‐11	AKT1,ATM,BCL2,BRCA1,CASP3,CASP8, CDKN1A,ERBB2,MDM2,PIK3CA,TP53
hsa04066	HIF‐1 signaling pathway	1.73E‐11	AKT1,BCL2,CDKN1A,CDKN1B,EGF,EGFR, EP300,ERBB2,IGF1R,MTOR,PIK3CA,VEGFA
hsa05216	Thyroid cancer	4.03E‐11	BRAF,CCND1,CDH1,CDKN1A,CTNNB1,HRAS, KRAS,MYC,TP53
hsa04934	Cushing's syndrome	1.42E‐10	AHR,APC,BRAF,CCND1,CDK2,CDK4,CDKN1A, CDKN1B,CTNNB1,CYP17A1,EGFR,GNAS,WNT10B
hsa04914	Progesterone‐mediated oocyte maturation	2.08E‐10	AKT1,AURKA,BRAF,CDC25A,CDK1,CDK2, IGF1R,KRAS,MAPK8,PGR,PIK3CA
hsa05211	Renal cell carcinoma	2.08E‐10	AKT1,BRAF,CDKN1A,EP300,HRAS,KRAS, PIK3CA,TGFA,TGFB1,VEGFA
hsa04630	Jak‐STAT signaling pathway	2.23E‐10	AKT1,BCL2,CCND1,CCND2,CDKN1A,EGF, EGFR,EP300,HRAS,MTOR,MYC,PIK3CA,PRLR
hsa04140	Autophagy ‐ animal	3.25E‐09	AKT1,BCL2,CTSD,HRAS,IGF1R,KRAS,MAPK8, MTOR,PIK3CA,PTEN,RB1CC1
hsa04926	Relaxin signaling pathway	4.68E‐09	AKT1,EGFR,FIGF,GNAS,HRAS,KRAS,MAPK8, PIK3CA,TGFB1,VEGFA,VEGFC
hsa04210	Apoptosis	6.65E‐09	AKT1,ATM,BCL2,CASP3,CASP8,CTSD,HRAS, KRAS,MAPK8,PIK3CA,TP53
hsa04550	Signaling pathways regulating pluripotency of stem cells	8.10E‐09	AKT1,APC,CTNNB1,FGFR2,HRAS,IGF1R, KRAS,MYC,PIK3CA,SMAD4,WNT10B

**TABLE 3:** KEGG pathway in fatty liver

Term ID	Term description	False discovery rate	Genes
hsa04932	Nonalcoholic fatty liver disease (NAFLD)	3.44E‐09	ADIPOQ,CYP2E1,IL6,INS,LEP,PPARA,SREBF1,TNF
hsa04152	AMPK signaling pathway	1.03E‐06	ACACA,ADIPOQ,FASN,INS,LEP,SREBF1
hsa04931	Insulin resistance	1.68E‐05	IL6,INS,PPARA,SREBF1,TNF
hsa04920	Adipocytokine signaling pathway	9.04E‐05	ADIPOQ,LEP,PPARA,TNF
hsa04910	Insulin signaling pathway	0.0009	ACACA,FASN,INS,SREBF1
hsa04930	Type II diabetes mellitus	0.0009	ADIPOQ,INS,TNF
hsa00061	Fatty acid biosynthesis	0.0029	ACACA,FASN
hsa04010	MAPK signaling pathway	0.0107	FGF21,INS,NLK,TNF
hsa04068	FoxO signaling pathway	0.0109	IL6,INS,NLK
hsa01523	Antifolate resistance	0.0117	IL6,TNF
hsa05143	African trypanosomiasis	0.0126	IL6,TNF
hsa05332	Graft‐versus‐host disease	0.0129	IL6,TNF
hsa04940	Type I diabetes mellitus	0.0138	INS,TNF
hsa04975	Fat digestion and absorption	0.0138	APOB,MTTP
hsa01212	Fatty acid metabolism	0.0171	ACACA,FASN
hsa05144	Malaria	0.0171	IL6,TNF
hsa05134	Legionellosis	0.0196	IL6,TNF
hsa00590	Arachidonic acid metabolism	0.0234	CYP2E1,GGT1
hsa05321	Inflammatory bowel disease (IBD)	0.0234	IL6,TNF
hsa03320	PPAR signaling pathway	0.0288	ADIPOQ,PPARA
hsa05133	Pertussis	0.0289	IL6,TNF
hsa04060	Cytokine‐cytokine receptor interaction	0.0321	IL6,LEP,TNF
hsa05410	Hypertrophic cardiomyopathy (HCM)	0.0321	IL6,TNF
hsa01100	Metabolic pathways	0.0322	ACACA,CYP2E1,FASN,GGT1,GPT,PNPLA3
hsa05323	Rheumatoid arthritis	0.0322	IL6,TNF
hsa04211	Longevity regulating pathway	0.0324	ADIPOQ,INS
hsa04657	IL‐17 signaling pathway	0.034	IL6,TNF
hsa04640	Hematopoietic cell lineage	0.0341	IL6,TNF
hsa05146	Amoebiasis	0.0341	IL6,TNF
hsa04066	HIF‐1 signaling pathway	0.0344	IL6,INS
hsa04620	Toll‐like receptor signaling pathway	0.0344	IL6,TNF
hsa04922	Glucagon signaling pathway	0.0344	ACACA,PPARA
hsa04933	AGE‐RAGE signaling pathway in diabetic complications	0.0344	IL6,TNF
hsa05142	Chagas disease (American trypanosomiasis)	0.0344	IL6,TNF
hsa04668	TNF signaling pathway	0.0354	IL6,TNF
hsa04151	PI3K‐Akt signaling pathway	0.0418	FGF21,IL6,INS
hsa05160	Hepatitis C	0.0481	PPARA,TNF

### 3.2 TAM inhibits the proliferation of breast cancer cells

**FIGURE 3:**
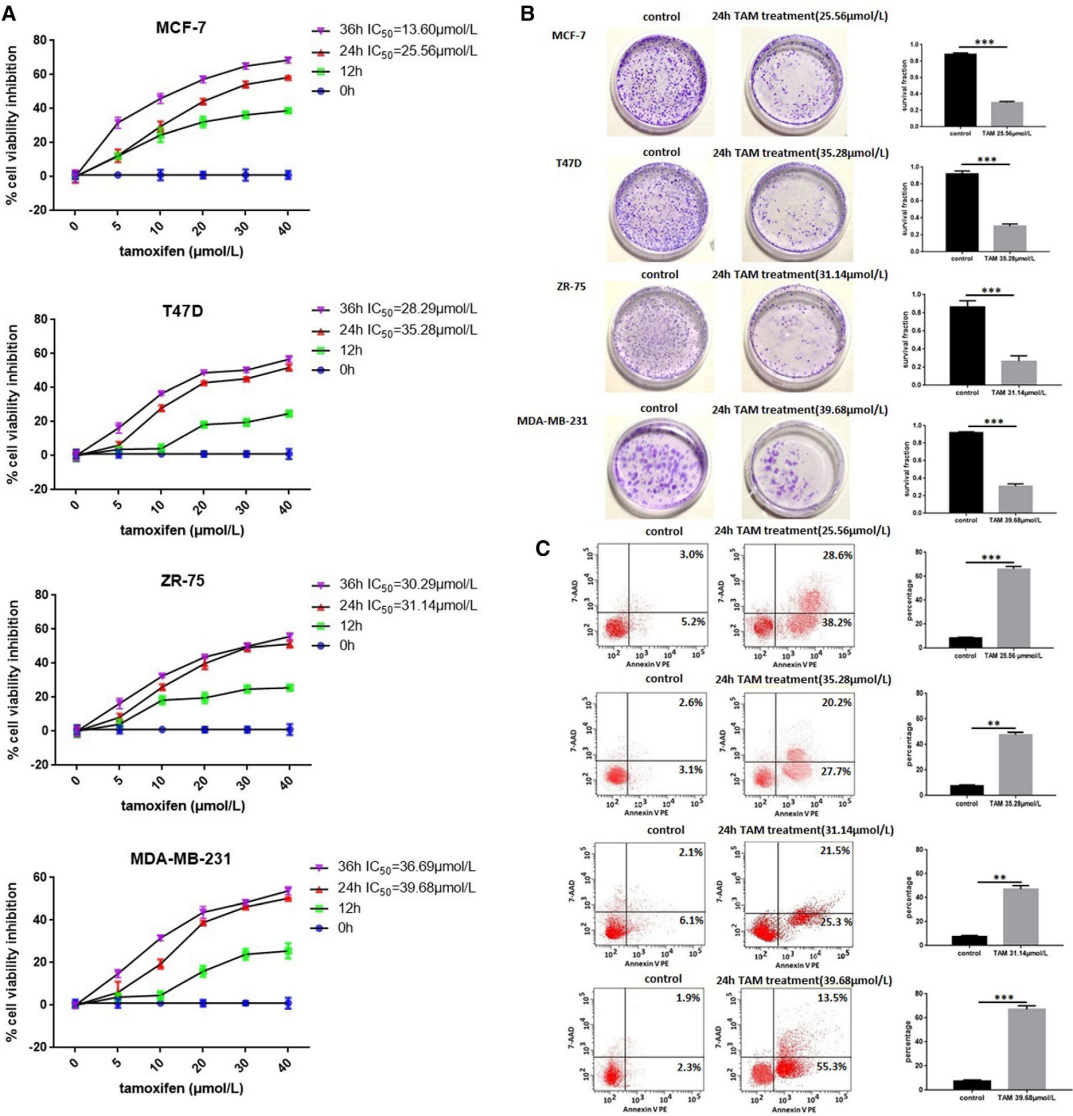
A, TAM decreased the growth of breast cancer cell lines (MCF‐7, T47D, ZR‐75, and MDA‐MB‐231) in a dose‐ and time‐dependent manner. B, The effect of TAM on clone formation capability of breast cancer cells. C, TAM‐induced apoptosis of breast cancer cells. **P < .01, ***P < .001

The effects of TAM on the viability of breast cancer cells were evaluated. We found that TAM decreased the growth of breast cancer cell lines (MCF‐7, T47D, ZR‐75, and MDA‐MB‐231) in dose‐ and time‐dependent manners (Figure 3A). Limited inhibitory effects on MCF‐7, T47D, ZR‐75, and MDA‐MB‐231 were observed even when the TAM concentrations were 25.56, 35.28, 31.14, and 39.68 µmol/L (IC50), respectively. These results indicate that TAM inhibits the growth of breast cancer cells at concentrations more than 25.56 µmol/L.

### 3.3 TAM inhibits clone formation and induces apoptosis of breast cancer cells

We determined the effects of TAM on the clone formation capability of breast cancer cells (MCF‐7, T47D, ZR‐75, and MDA‐MB‐231). Treatment with TAM markedly decreased the number of colonies compared to untreated cells (Figure 3B). Treatment of breast cancer cells with TAM caused an increase in apoptotic cells compared to untreated breast cancer cells (Figure 3C). These results demonstrate that TAM has potent effects against clone formation and induces the apoptosis of breast cancer cells.

### 3.4 TAM induces lipid accumulation in LO2 Cells

**FIGURE 4:**
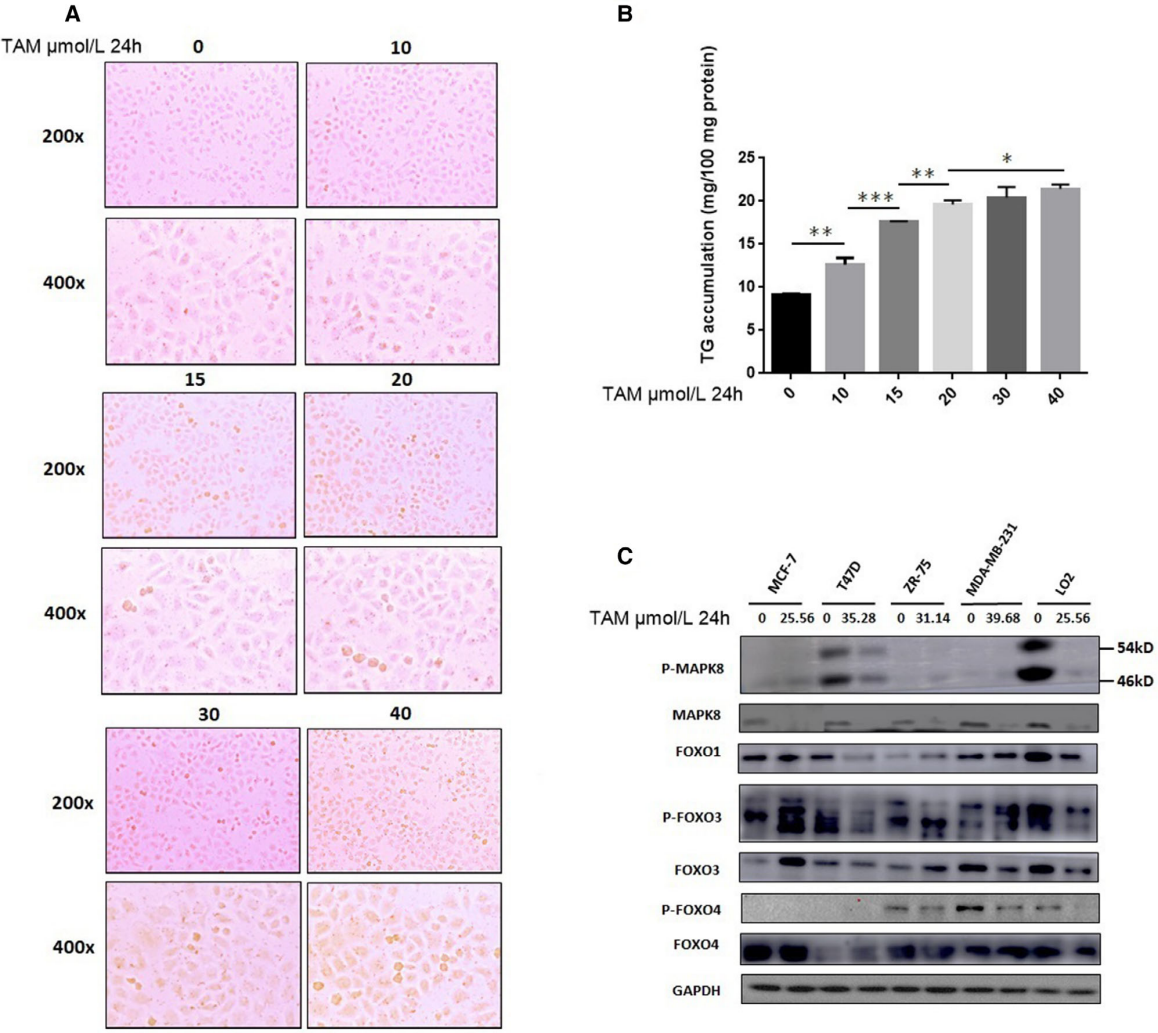
A, TAM‐induced hepatocyte steatosis in LO2 cells. B, Significant increases in TG were observed in LO2 cells treated with ≥10 µmol/L of TAM. C, Different transmission of MAPK8/FoxO signaling pathway in breast cancer cells (MCF‐7, T47D, ZR‐75, and MDA‐MB‐231) and liver cells (LO2) exposed to TAM. *P < .05, **P < .01, ***P < .001

We treated LO2 cells with various concentrations of TAM for 24 hours. Lipid accumulation was examined after Oil Red O staining. As shown in Figure 4A, TAM induced hepatocyte steatosis in LO2 cells, and cells treated with TAM accumulated significant amount of lipid droplets in a dose‐dependent manner. Consistently, measurements of TG concentration in cell lysates showed that significant increases in TG were observed in LO2 cells treated with ≥10 µmol/L TAM (Figure 4B).

### 3.5 TAM induces FLD by disrupting the MAPK8/FoxO signaling pathway

As shown in Figure 4C, as an upstream mediator of FoxO signaling, MAPK8 was suppressed in breast cancer cells (MCF‐7, T47D, ZR‐75, and MDA‐MB‐231) and liver cells (LO2) treated with TAM. Because of the variable differences between cell lines, the expression levels of FoxO proteins changed differently in breast cancer cell lines. When treated with TAM, p‐FOXO3 and FOXO3 were up‐expressed in MCF‐7 cells; FOXO1 and p‐FOXO3 were down‐expressed in T47D cells; FOXO4 was up‐expressed in T47D cells; FOXO1, p‐FOXO3, and FOXO3 were up‐expressed in ZR‐75 cells; p‐FOXO3 and FOXO4 were up‐expressed in MDA‐MB‐231 cells; and FOXO3 and p‐FOXO4 were down‐expressed in MDA‐MB‐231 cells. And all proteins in liver cells (LO2) were down‐expressed when exposed to TAM. These results indicate that TAM induces FLD by disrupting the MAPK8/FoxO signaling pathway in patients with breast cancer.

## 4 DISCUSSION

Breast cancer is the most common and aggressive cancer among women worldwide. TAM has been the gold standard treatment for all stages of estrogen receptor (ER)‐positive breast cancer, and it is also effective against ER‐negative breast cancer. However, TAM is associated with an increased risk of the development of FLD,^27^ and studies have reported that about 43% of breast cancer patients using TAM may develop FLD within the first 2 years,^27^, ^28^, ^29^ indicating the need to manage fatty liver with a positive strategy through early prevention. It is very urgent to find an effective paradigm for clarifying the functional mechanism underlying breast cancer and TAM‐induced fatty liver.

In this study, we used a combination of bioinformatics analysis and conventional experiments to clarify the functional mechanisms underlying breast cancer and TAM‐induced FLD. Bioinformatics analysis was done as follows: (a) DPTs of TAM were identified by DrugBank5.1.7; (b) significant genes in breast cancer and fatty liver were identified by MalaCards; (c) KEGG pathways of those significant genes were analyzed using STRING; and (d) KEGG Mapper analysis was performed. We found that MAPK8 was one DPT of TAM, and significant genes of breast cancer and fatty liver were correlated with the MAPK and FoxO signaling pathways; the MAPK signaling pathway was found to be upstream of the FoxO signaling pathway. The functional relevance of breast cancer and TAM‐induced fatty liver was validated by the experimental data. We verified that TAM may induce fatty liver in breast cancer through the MAPK8/FoxO signaling pathway.

MAPK8, also known as c‐Jun NH2‐terminal kinase‐1 (JNK1), is a member of the MAPK family.^30^ Studies overexpressing a DN JNK1 mutant have demonstrated that TAM can stimulate JNK1 activity and interfere with the JNK pathway.^31^, ^32^ Furthermore, it has been reported that TAM induces apoptosis of breast cancer cells through the JNK1 pathway.^33^ Sabio et al^34^ reported that JNK1 serves to prevent hepatic steatosis. Consistently, our study found that MAPK8 was a DPT of TAM (Table 1), which induces the apoptosis of breast cancer cells (Figure 3C) and steatosis in liver cells (Figure 4).

The FoxO family, which consists of FoxO1, FoxO3, FoxO4, and FoxO6, is known as a tumor suppressor that limits cell proliferation and induces apoptosis.^35^ However, paradoxical roles of FoxO proteins in cancer progression were recently described^36^; for example in acute and chronic myeloid leukemia, FoxO proteins maintain leukemia‐initiating cells. These factors may also promote the invasion of breast cancer,^37^ and FoxO proteins contribute to treatment resistance in multiple cases, including targeted therapies.^38^ Hornsveld et al^39^ reported that FoxO proteins both suppress and support breast cancer progression. Dong^40^ claimed that FoxO proteins play critical roles in maintaining metabolic and cellular homeostasis in the liver, and their suppression may be involved in NAFLD development. In our study, we found that TAM can both upregulate and downregulate FoxOs and P‐FoxOs in different breast cancer cell lines (MCF‐7, T47D, ZR‐75, and MDA‐MB‐231), which may predict different prognosis to types of breast cancer. Meanwhile, TAM downregulated FoxOs in the LO2 liver cell line, which may induce FLD.

As determined using integrated bioinformatics analysis, the MAPK8/FoxO signaling pathway is important for the development of cancer and fatty liver. We confirmed that TAM can function through the MAPK8/FoxO signaling pathway in breast cancer cells (MCF‐7, T47D, ZR‐75, and MDA‐MB‐231) and liver cells (LO2). Thus, we predict that TAM induces fatty liver by interfering with the MAPK8/FoxO signaling pathway. However, further studies such as siRNA or shRNA directed against DPT (MAPK8) are urgently warranted to validate the prediction, and further mechanisms would be uncovered.

## 5 CONCLUSIONS

In summary, combined bioinformatics analysis and experimental verification provided an effective and convenient approach for clarifying the molecular mechanism underlying TAM‐induced FLD in breast cancer patients. Using existing drug and disease databases as the BioGPS, leading researchers combine web‐based resources and experimental results with clinical application. This novel comprehensive research approach can be used to determine the molecular mechanism underlying the complicating effects of drugs in cancer treatment.

## CONFLICT OF INTEREST

The authors declare no conflict of interest.

## Data Availability

The data and materials used in the current study are available from the corresponding author on reasonable request.
