# Erratum for the Clinical and Translational Medicine “Tamoxifen induces fatty liver disease in breast cancer through the MAPK8/ FoxO pathway” by Liuyun Gong et al.

**DOI:** 10.1002/ctm2.1807

**Published:** 2024-08-16

Liuyun Gong, Hanmin Tang, Zhenzhen Luo, Xiao Sun, Xinyue Tan, Lina Xie, Yutiantian Lei, Mengjiao Cai, Chenchen He, Jinlu Ma, Suxia Han

Department of Oncology, The First Affiliated Hospital, Xi'an Jiaotong University, Xi'an, Shanxi 710061, P. R. China.

Following the publication of the original article,^1^ the authors identified minor errors in Figure 4A, where the images of some groups were incorrect. Because during image assembly, we took 3–5 pictures at random from different angles of each slide, we mistakenly used the pictures. In Figure 4A: An overlap between 20 μmol/L TAM (200x) and 30 μmol/L TAM (400x) groups, we found our mistakes and reassembled the images, we also corrected the images for each group, we want to make a published erratum. More importantly, we promise that the erratum has no impact on the conclusion and description of the article.

1. Gong LY, Tang HM, Luo ZZ, et al. Tamoxifen induces fatty liver disease in breast cancer through the MAPK8/FoxO pathway. *Clin Transl Med*. **2020**;10(1):137‐150. Article. https://doi.org/10.1002/ctm2.5.

**ORIGINAL FIGURE 4:**
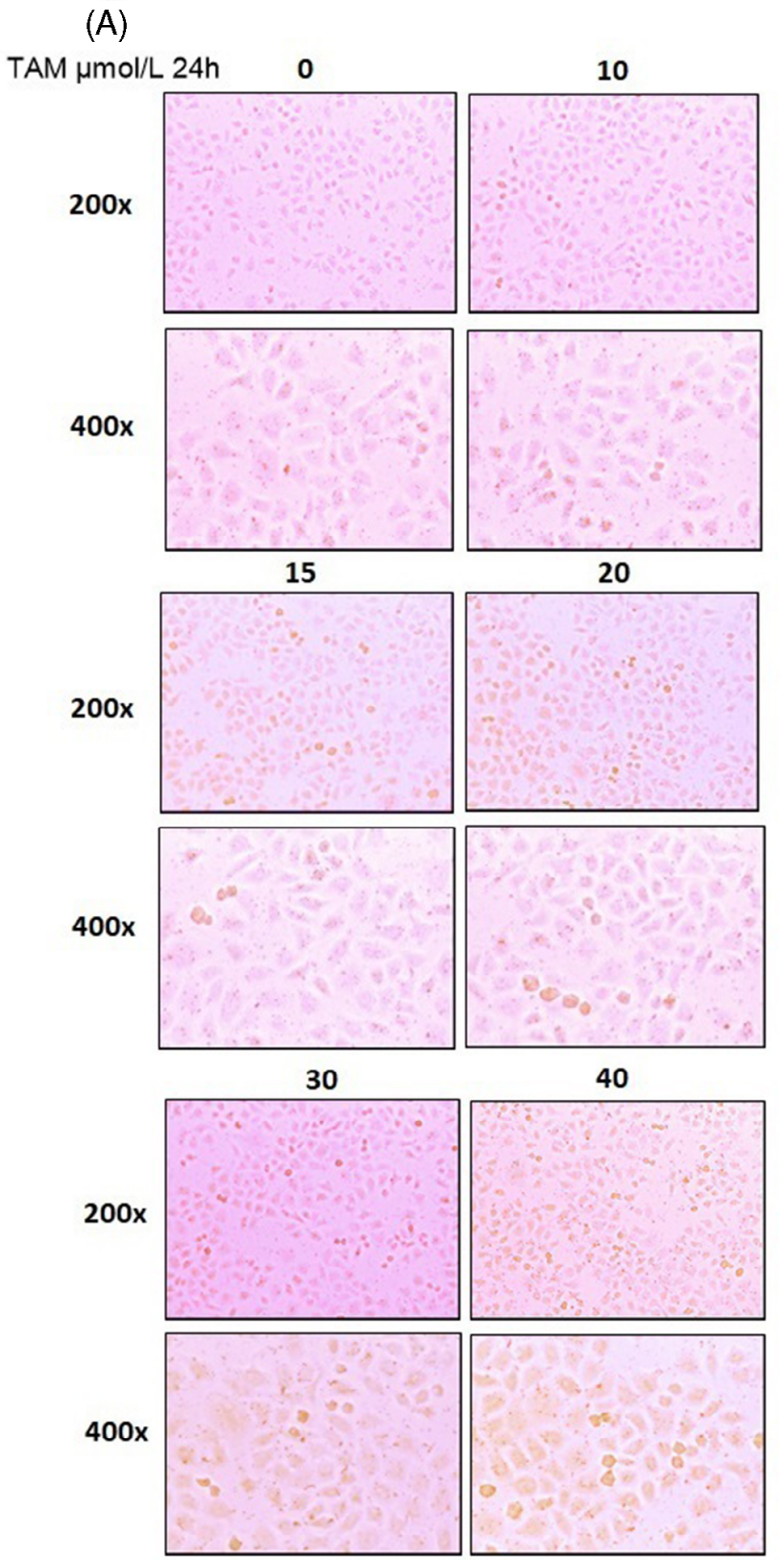
(A) TAM‐induced hepatocyte steatosis in LO_2_ cells.

**UPDATED FIGURE 4:**
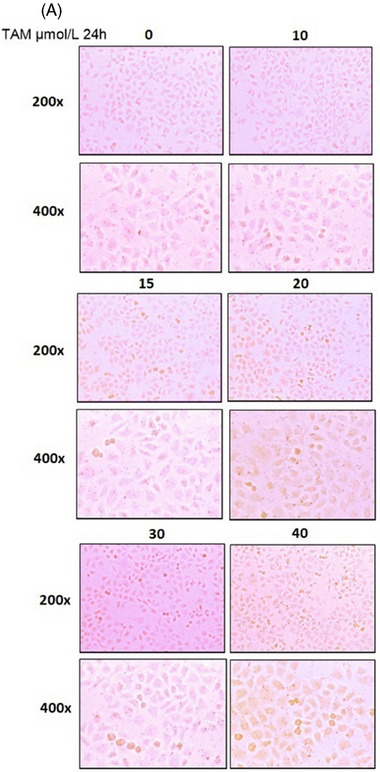
(A) TAM‐induced hepatocyte steatosis in LO_2_ cells.

